# Genetic profiling of human bone marrow and adipose tissue-derived mesenchymal stem cells reveals differences in osteogenic signaling mediated by graphene

**DOI:** 10.1186/s12951-021-01024-x

**Published:** 2021-09-22

**Authors:** Amber F. MacDonald, Ruby D. Trotter, Christopher D. Griffin, Austin J. Bow, Steven D. Newby, William J. King, Lisa L. Amelse, Thomas J. Masi, Shawn E. Bourdo, Madhu S. Dhar

**Affiliations:** 1 College of Veterinary Medicine, University of Tennessee, Knoxville, TN 37996 USA; 2 Center for Integrative Nanotechnology Sciences, University of Arkansas at Little Rock, Little Rock, AR 72204 USA; 3 University of Tennessee Graduate School of Medicine, Knoxville, TN 37996 USA

**Keywords:** Human mesenchymal stem cells, Osteogenesis, Focused arrays, Osteogenic signaling, Angiogenic signaling

## Abstract

**Background:**

In the last decade, graphene surfaces have consistently supported osteoblast development of stem cells, holding promise as a therapeutic implant for degenerative bone diseases. However, until now no study has specifically examined the genetic changes when stem cells undergo osteogenic differentiation on graphene.

**Results:**

In this study, we provide a detailed overview of gene expressions when human mesenchymal stem cells (MSCs) derived from either adipose tissue (AD-MSCs) or bone marrow (BM-MSCs), are cultured on graphene. Genetic expressions were measured using osteogenic RT^2^ profiler PCR arrays and compared either over time (7 or 21 days) or between each cell source at each time point. Genes were categorized as either transcriptional regulation, osteoblast-related, extracellular matrix, cellular adhesion, BMP and SMAD signaling, growth factors, or angiogenic factors. Results showed that both MSC sources cultured on low oxygen graphene surfaces achieved osteogenesis by 21 days and expressed specific osteoblast markers. However, each MSC source cultured on graphene did have genetically different responses. When compared between each other, we found that genes of BM-MSCs were robustly expressed, and more noticeable after 7 days of culturing, suggesting BM-MSCs initiate osteogenesis at an earlier time point than AD-MSCs on graphene. Additionally, we found upregulated angiogenic markers in both MSCs sources, suggesting graphene could simultaneously attract the ingrowth of blood vessels in vivo. Finally, we identified several novel targets, including distal-less homeobox 5 (*DLX5*) and phosphate-regulating endopeptidase homolog, X-linked (*PHEX*).

**Conclusions:**

Overall, this study shows that graphene genetically supports differentiation of both AD-MSCs and BM-MSCs but may involve different signaling mechanisms to achieve osteogenesis. Data further demonstrates the lack of aberrant signaling due to cell-graphene interaction, strengthening the application of specific form and concentration of graphene nanoparticles in bone tissue engineering.

**Graphic abstract:**

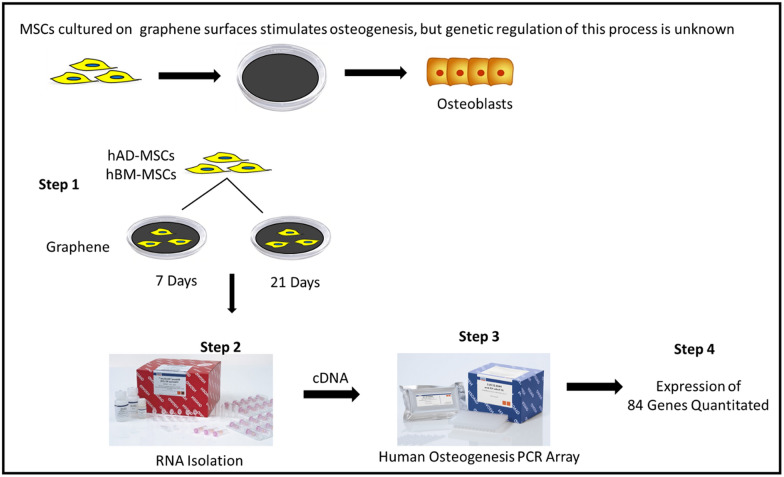

## Background

In the United States, there are approximately 1 million new cases of severe bone defects that require medical intervention. Traditionally these defects are filled with autologous bone grafts, in which bone removed from the hip or ribs is implanted into the defected area. Unfortunately, this method causes many limitations as the procedure alone is highly invasive, increases the risk of infection, causes donor site morbidity, and overall is not appropriate for geriatric patients. An alternative and extensively investigated medical strategy is bone tissue engineering. Bone tissue engineering requires viable or osteoprogenitor cells and natural/synthetic biomaterials which together are used in the fabrication of novel scaffold constructs [1]. Biomaterials developed for bone are manufactured with specific functions: (1) to deliver and home stem cells to the injury site, (2) to induce osteoblast differentiation of the externally delivered osteoprogenitors cells, (3) to induce osteoblast differentiation of the endogenous progenitor cells, and (4) should be mechanically strong, flexible, and gradually resorb as new bone is formed over time.

Since graphene’s discovery in 2004, graphene-based nanocomposite scaffolds have gained significant appreciation in biomedicine, specifically bone tissue engineering. Graphene is a single isolated layer of graphite, having a two-dimensional structure consisting of carbon atoms orchestrated as hexagonal rings. It has been called “the wonder material” due to its superthin, yet super-strong and flexible features. In addition to single layer graphene, few-layer graphene can also be utilized for many of the same applications that single-layer graphene has been touted. Many variations of graphene have been developed that differ largely on the oxygen content—from pristine with little to no oxygen in the carbon network to graphene oxide (GO) with the highest amounts of incorporated oxygen. There are many terms that can refer to variation in the chemical makeup, including reduced graphene oxide (rGO) and highly reduced graphene oxide (hrGO). These modifications are necessary for many applications since the pristine form of graphene is hydrophobic and consequently, cannot be dissolved or readily dispersed in water or bodily fluids. Aside from simple oxygen functionalization, many other functionalities and treatments can be incorporated to make graphene highly dispersible and less toxic [2–5]. We have coined distinct terms for the oxygen functionalized graphene based on the oxygen content, i.e. low and high oxygen content graphene as LOG and HOG, respectively [6, 7].

Graphene was first recognized to be biocompatible and a potential bone biomaterial in 2010 after recognizing that human osteoblasts and mesenchymal stem cells (MSCs) adhered and proliferated on graphene better than on silicon dioxide substrates [8]. Since then, multiple laboratories (including ours) have recognized graphene and its derivatives as valid osteoinductive and osteoconductive nanomaterials in vitro and in vivo [9, 10]. Our research group has demonstrated that a low-oxygen content graphene (LOG) material was cytocompatible and supported the adherence, proliferation and osteogenic differentiation of goat bone marrow derived MSCs (BM-MSCs) in vitro [9]. We then confirmed the osteoinductive and osteoconductive potential of LOG with goat adipose derived MSCs (AD-MSCs) in vivo [10]. Likewise, we most recently demonstrated that LOG exhibited similar effects on human AD-MSCs in vitro, i.e. MSCs underwent osteogenic differentiation without any chemical induction. Human MSCs exposed to graphene surfaces expressed specific integrin heterodimers and the corresponding ECM proteins, suggesting that the structure and topography of LOG surface potentially induces the expression of bone-specific ECM and thus, promotes MSCs to undergo osteogenic differentiation [11].

Even though graphene-based nanomaterials are being used in bone tissue engineering and their biological role in osteoblast differentiation of MSCs has been demonstrated in multiple studies, in vitro and in vivo, the signals that are triggered i.e. the knowledge of the signal transduction pathways that the cells undergo during this process is limited [12, 13].

Adult MSCs can be isolated from a variety of tissues including, bone marrow, adipose tissue, dental pulp, umbilical cord blood, Wharton’s jelly, and the placenta. Even though bone marrow and adipose tissue are the most commonly used tissue sources of MSCs, their efficacy in regenerative medicine is varied. This is primarily due to the donor-to-donor variation as well as the variations in isolation and in vitro cell culturing protocols of expansion [14–16]. The application of either bone marrow or adipose tissue-derived MSCs in bone tissue engineering can be further affected by the interaction between MSCs and the nanomaterials used. Hence, in order to assess the efficacy of nanomaterial/cell constructs and to improve the fabrication of the nanomaterials, it is important to evaluate differences in cell signaling in presence of the nanomaterials.

This study was carried out to understand the genetic expressions that graphene regulates on human MSCs i.e. to identify molecular targets that specifically communicate osteogenic differentiation of MSCs. The study design also provided us with the opportunity to compare and contrast the osteogenic response between bone marrow and adipose tissue-derived MSCs. All graphene and MSC studies report conclusions from single reactions of osteoblastic markers [17–19], but this method provides minuscule insight on how graphene nanomaterials influence cell behavior during osteogenesis. Therefore, the objective of this study was to measure and compare changes in gene expression during osteogenesis of human MSCs derived from adipose tissue and bone marrow on a LOG surface. Based on previous literature and data from our laboratory, we hypothesized that using osteogenic focused arrays and monitoring changes in osteogenesis over a specific time period, we will be able to evaluate the key pathways that MSCs go through in presence of functionalized form of graphene.

## Materials and methods

### Tissue procurement, cell isolation and characterization

Human adipose tissue-derived MSCs were isolated, characterized and cryobanked as described earlier [11]. Prior to cell isolation, patient consent was obtained and approved by an IRB protocol at the University of Tennessee Medical Center in Knoxville. Adipose tissue was collected from patients undergoing pannulectomies. Following cell expansion, human adipose-derived MSCs (AD-MSCs) were confirmed for cell morphology, protein markers, and trilineage differentiation, as described earlier [11, 20].

Human bone-marrow derived MSCs (BM-MSCs) were commercially purchased from American Type Culture Collection (ATCC) (Manassas, VA). Cells were expanded and cryopreserved as per ATCC’s recommendations. MSCs were confirmed for their adherence to tissue culture plastic and ability for tri-lineage differentiation in vitro.

Cells from passages 2–6 were used in all experiments described.

### Preparation and characterization of graphene

#### Processing conditions

Pristine graphene was purchased commercially from Angstron Materials (Dayton, Ohio) and oxidized within an acidic mixture (6:2:3 ratio of sulfuric acid, nitric acid, and water) as described earlier [6, 10]. The final product was a low-oxygen functionalized form of graphene (LOG) with approximately 6–10% oxygen content and was confirmed to be the form used in previous studies [6, 10]. LOG was dispersed in ethanol/water by sonication. Aliquots of the dispersion were used to coat the dishes for cell culture.

#### Deposition of graphene

Non-tissue cultured treated dishes were coated with LOG to produce uniform surfaces with very little exposed plastic. For all experiments, the LOG concentration was 0.2 mg/cm^2^ of dish surface.

#### Surface topography

Surface roughness/topography was investigated using atomic force microscopy (AFM). A Bruker Dimension AFM using a Budget Sensors Tap300Al-G tip (300 kHz and 40 N/m) in tapping mode. Random spots were chosen for analysis on a 100 mm petri dish and scan sizes of 50 mm × 50 mm were collected. An average of 7 spots and standard deviation was determined. NanoScope Analysis 1.5 (Bruker) software was used to analyze the surface images to determine average roughness (Ra) and root-mean-square (Rq).

### Osteogenesis and mineralization of MSCs

AD-MSCs and BM-MSCs were grown to 70–80% confluency in growth media (DMEM F12 + 10%FBS + 1%penicillin–streptomycin-antimycotic) and incubated in an atmosphere of 5% CO_2_ at 37 °C. For experimental conditions, cells were harvested with 0.05% trypsin and seeded at 1 × 10^5^/well of a 12 well plate and 1 × 10^6^/100 mm cell culture dish coated with LOG. Cells were cultured on LOG for either 7 or 21 days and were maintained in growth media without any osteo-differentiation inducers throughout the study. At specified time points, cells were either stained with Alizarin red and quantitated as reported earlier [11] or collected for RNA experiments (described below).

To ensure that AD-MSCs generated in our lab and commercial BM-MSCs retained their osteogenic potential under standard conditions, cells were cultured on tissue culture polystyrene surface in presence of osteogenic induced medium (growth media supplemented with 10 nM β-glycerophosphate, 100 nM dexamethasone, and 155 µM ascorbic acid). Osteogenesis was confirmed by Alizarin red staining and quantitation as previously described [11].

### RNA isolation

Cells were detached from LOG with 0.05% trypsin for approximately 40 min. followed by centrifugation. A cell pellet was combined from two-100 mm LOG coated dishes to ensure the RNA quantity was sufficient for triplicate PCR reactions. Total RNA was isolated using an RNeasy® Mini Kit following the manufacturer’s instructions (Qiagen, Germantown, MD, #74104). To measure RNA purity and quantity, samples were loaded onto a Take3 plate, and read on Epoch microplate spectrophotometer (BioTek Instruments, Winooski, VT) with Gen5 version 2.09 software. The 260/280 nm absorbance ratio determined RNA purity and was considered optimal at approximately 2.0. RNA 6000 Nano Kit and the 2100 Bioanalyzer system was used to evaluate the integrity as per the manufacturer’s recommendations (Agilent Technologies, Santa Clara, CA) [21].

### Human osteogenesis PCR array

RT^2^ Profiler PCR Human Osteogenesis Array (Qiagen, Hilden, Germany, #PAHS-026Z) was used to evaluate differentially expressed genes from AD-MSCs and BM-MSCs cultured on LOG. 1 µg of RNA was reversed transcribed to cDNA with a RT^2^ First Strand Kit (Qiagen, Hilden, Germany, #330401). The cDNA was added into a RT^2^ SYBR Green Mastermix (Qiagen, Hilden, Germany, #330524) before loading 25 µL (~ 10.4 ng) per well. cDNA synthesis and PCR reactions were performed according to the manufacturer’s recommendations [22].

### Statistical analysis

Gene expressions from CT values were analyzed using Qiagen Gene Globe software to determine the relative fold change (https://geneglobe.qiagen.com/us/analyze/). In the first analyses, gene expression data obtained from AD-MSCs cultured on tissue culture polystyrene surface in presence of osteogenic induced medium (growth media supplemented with 10 mM β-glycerophosphate, 100 nM dexamethasone, and 155 µM ascorbic acid) for 21 days was set as the control. MSCs cultured on the LOG surface without the differentiation cocktail for 21 days was designated as the test group. Thereafter, all comparisons were carried out between the AD and BM-MSCs cultured solely on the LOG surface.

To evaluate cell signaling on LOG surface, we compared the changes in gene expression for each cell type between days 7 and 21. Expression at day 7 was set as the control and day 21 was designated as the test group. Subsequently, the two cell types were compared at each time point, with AD-MSCs set as the control group and BM-MSCs as the tested group. All comparisons were normalized using β-2 microglobulin (B2M) and glyceraldehyde-3-Phosphate Dehydrogenase (GAPDH) as the housekeeping genes. Data is shown from triplicate experiments, with fold changes statistically significant at *p* < 0.05.

### Cytoscape analyses of potential protein targets

Genes of interest were imported from the appropriate tables into Cytoscape software (https://cytoscape.org/) containing a basal nodal network derived from the updated BioGrid data set for Homo sapiens (https://thebiogrid.org/). The complete network was then filtered based on the target genes resulting in the input gene set nodes with residual connective line elements from the basal map. Genes were then sorted based on associated functional group and graphed onto propellor plot diagrams depicting the up and downregulated candidates for both experimental sample comparisons. Gene sets displayed in propellor plots serve to demonstrate the comparative difference in gene expression and thus, can be translated into protein–protein interactions for these two experimental groups as compared to a common control.

### Cytoskeletal organization and expression of ECM proteins

Cytoskeletal organization and morphology of BM-MSCs were assessed by evaluating the pattern of F-actin staining using previously reported methods [11]. The expression patterns of ECM proteins corresponding to the gene targets identified for the BM-MSCs were assessed qualitatively by immunofluorescence detection assays. The assessments were made during cell attachment (i.e. within 24 h of seeding) and osteogenic differentiation (21 days after seeding) Fibronectin 182, and collagen I, were evaluated as described earlier [11].

## Results

### Graphene nanomaterials display 6–10% oxygen content

Graphene surfaces have been extensively characterized and reported in previous publications [6, 7]. The material is distinct from the commercially available forms of pristine graphene and graphene oxide (GO) in oxygen content. It may share similar characteristics with reduced-GO or highly reduced-GO, however, we use the term low-oxygen graphene (LOG) since it is produced directly from commercially obtained pristine graphene powder, and not via the reduction of GO.

**Fig. 1:**
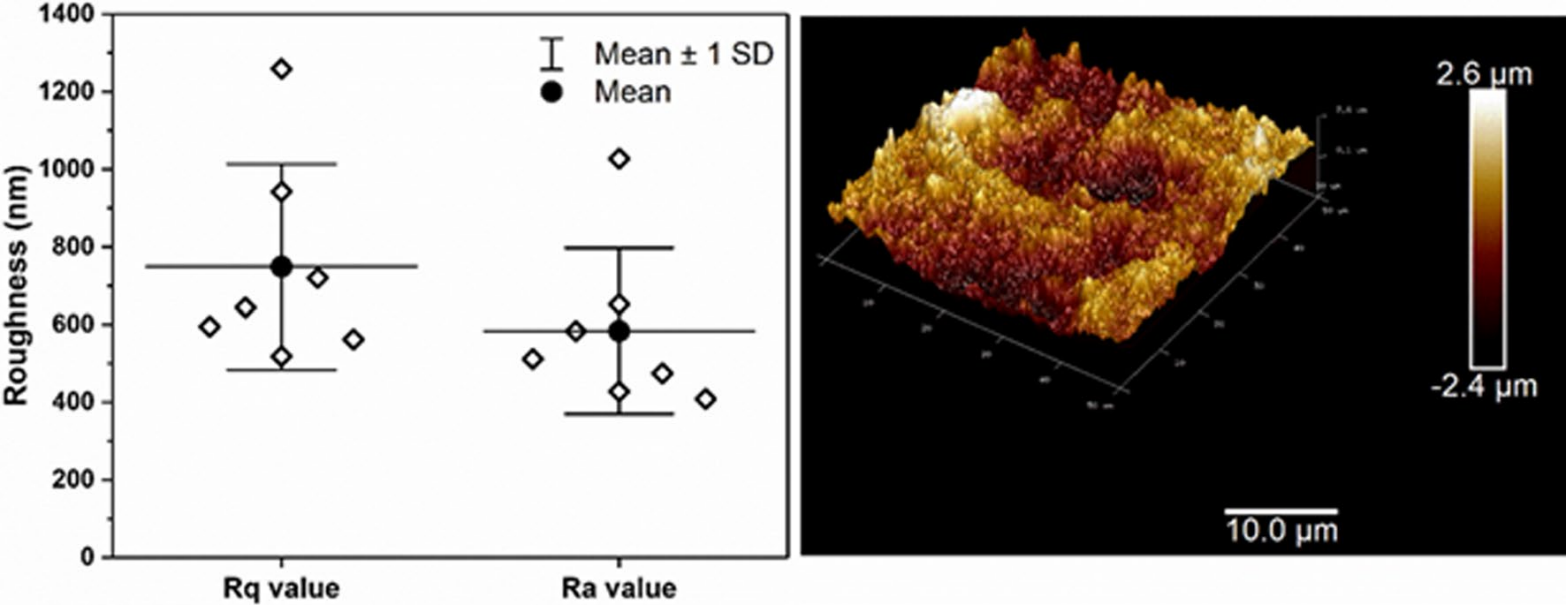
Atomic force microscopy. (Left panel) Plot of roughness values (Rq and Ra) from 7 AFM imaged: data shown with diamonds, mean value with solid circle + line, and the standard deviation with whiskers. (Right panel) reperesentative AFM image (approximately the average Rq and Ra values) from a random spot surface on 100 mm plastic petri dish

LOG preparations were consistent with that reported earlier, and contain 6–10% oxygen content, with trace (< 0.5%) amounts of sulfur and nitrogen [9, 10]. The oxygen moieties are distributed within the hydroxyl, carbonyl, ether, and carboxyl groups as reported in functionalized graphene with higher oxygen content, such as GO [6]. In addition to the surface chemistry of LOG, surface roughness, which is an important aspect for cell adhesion/attachment was evaluated [23, 24]. Figure 1 displays the root mean squared (R_q_) and average (R_a_) roughness values as determined from atomic force microscopy (AFM). The images collected from AFM show a rough surface topography providing numerous sites for possible cell attachment. Data is consistent with that reported earlier [6, 11].

### Human adipose tissue and bone marrow-derived MSCs display similar osteogenic behavior on LOG surface

**Fig. 2:**
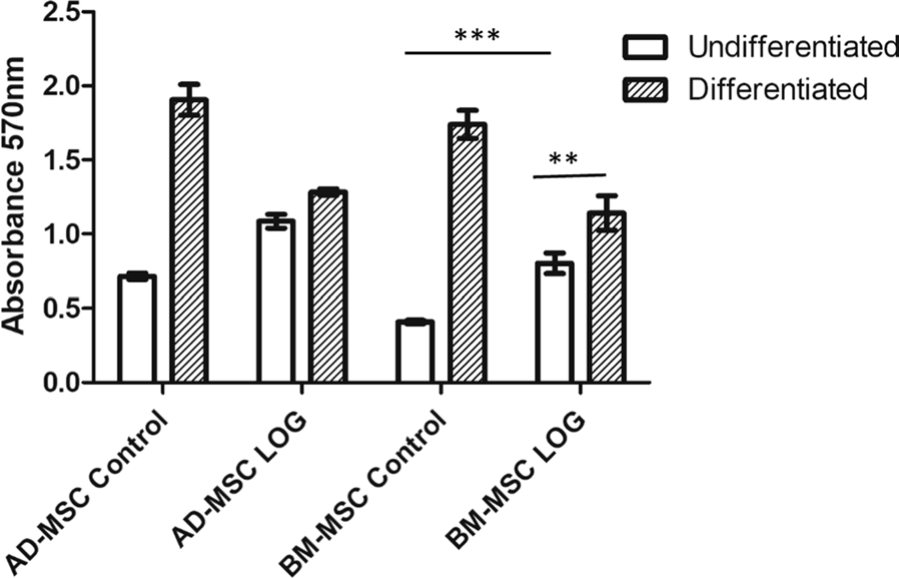
Osteogenic differentiation assay. AD-MSCs or BM-MSCs were seeded on tissue culture polystyrene (control) or LOG and cultured in either undifferentiated media (without osteogenic induction) or differentiated media (with osteogenic induction) for 21 days. Cells were then exposed to Alizarin red staining and read at absorbance 570 nm for calcium quantitation. Statistical significance (p < 0.05) is indicated by asterisks

We have previously reported that human and goat adipose tissue-derived MSCs undergo spontaneous osteogenesis on LOG without any chemical induction [9–11]. We have also demonstrated that goat adipose tissue and bone marrow-derived MSCs undergo osteogenesis using two distinct signaling pathways [25]. In view of these data, we evaluated and compared the osteogenic differentiation and mineralization of human BM-MSCs to AD-MSCs on LOG surfaces using Alizarin red staining and quantitation (Fig. 2). The calcium content as judged by Alizarin red staining was significantly greater in human BM-MSCs seeded on LOG surfaces relative to the cells on the tissue culture polystyrene surface (*p* < 0.0001). Interestingly, this upregulation was similar to that reported earlier for human AD-MSCs [11] and was observed in the absence of any osteogenic inducers. Calcium content was enhanced (*p* < 0.0025) when osteogenic inducers were added to the media. Results suggest that irrespective of the source, the LOG surface induces similar accumulation of calcium in both the adipose tissue and bone marrow-derived MSCs in vitro.

Surprisingly, in presence of the differentiation media + LOG, there was a decrease in the calcium content relative to the cells on tissue culture polystyrene surface. The reason for this outcome is unknown, and is beyond the scope of this study. Based on published literature, dexamethasone, beta glycerophosphate and ascorbic acid regulate several signal transduction pathways and hence, this effect should be investigated in future studies [26]. Therefore, in the current study, we sought to identify the molecular targets involved in LOG-mediated stem cell signaling without osteogenic inducers. In the experiments described below, cells were maintained in growth media alone without any supplementation of osteogenic reagents.

### High quality RNA was obtained from MSCs

**Fig. 3:**
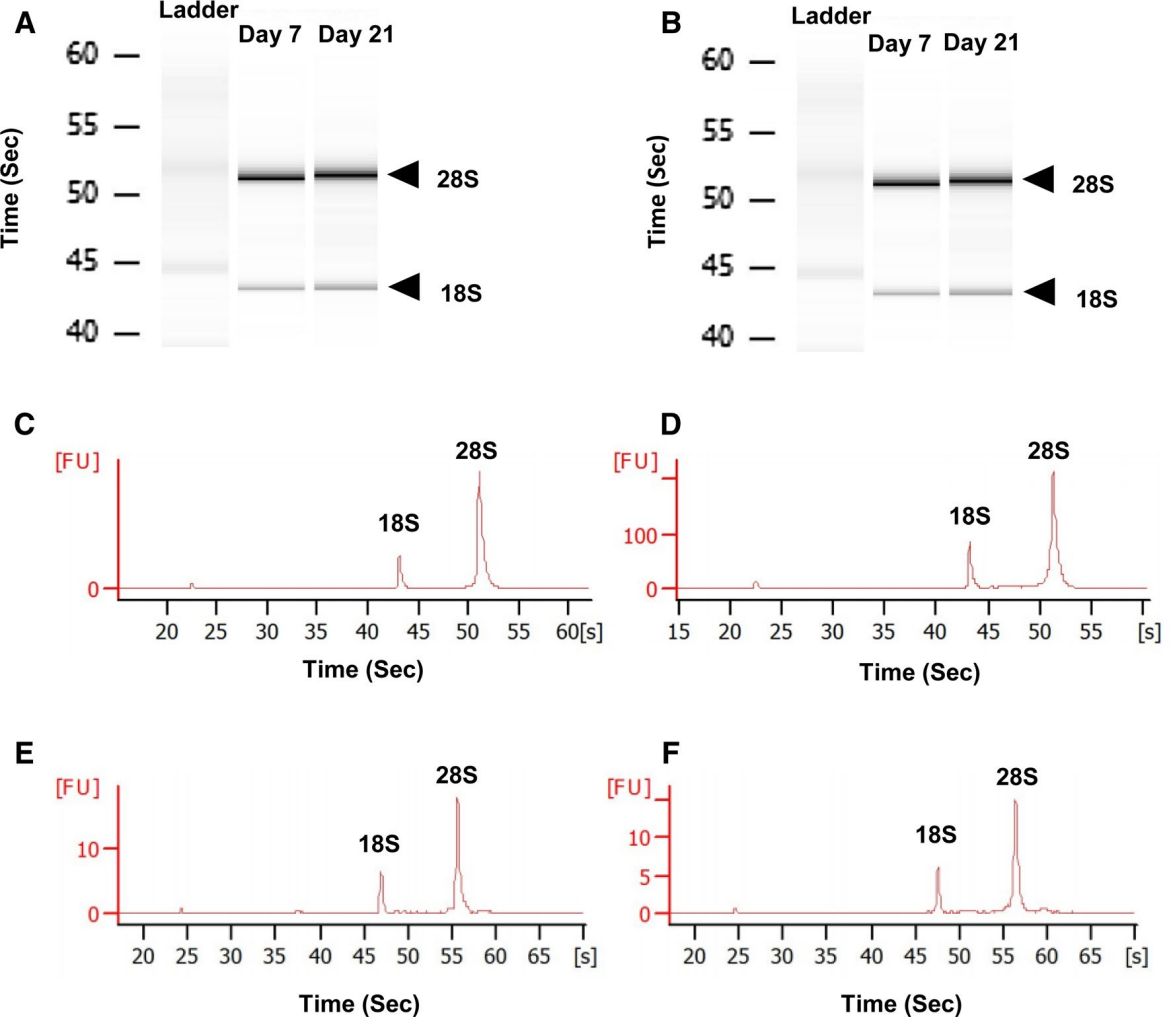
Assessment of RNA Quality. **A** Electrophoresis of total RNA from AD-MSCs cultured on LOG for either 7 or 21 days. **B** Electrophoresis of total RNA from BM-MSCs cultured on LOG for either 7 or 21 days. Arrows indicate bands of ribosomal subunits. **C** Electropherogram of AD-MSCs cultured on LOG for either 7 days (RIN = 9.10) or **D** 21 days (RIN = 9.60). **E** Electropherogram of BM-MSCs cultured on LOG for either 7 days (RIN = 9.40) or **F** 21 days RIN = 9.50

We harvested MSCs from LOG using trypsin, with longer than normal incubation time of about 40 min. As a result, we evaluated the RNA quantity and quality prior to PCR analyses. Electrophoresis of total RNAs from AD-MSCs and BM-MSCs in presence of LOG for both 7 and 21 days showed no degradation and intact ribosomal subunits, 18S and 28S bands (Fig. 3A, B). RNA quality was measured by RNA integrity number (RIN) ranging from 1–10, with RIN < 6 considered as a low quality sample [27]. An electropherogram confirmed high quality RNA with RIN values > 9.0 at both time points for both cell types (Fig. 3C–F.).

### Focused arrays to evaluate graphene-mediated differentiation

Osteogenesis is a complex signaling pathway coordinated by multiple gene and protein targets that mediate osteoblast differentiation of stem cells. Therefore, to understand osteogenic signaling stimulated by graphene, we evaluated gene expression patterns in human AD-MSCs and BM-MSCs using human osteogenesis focused PCR arrays (Qiagen, Hilden, Germany, #PAHS-026Z). These 96 well-arrays are coated with primers that target 84 genes of interest, 5 housekeeping genes for data normalization, and 7 controls to evaluate human genomic DNA contamination, performance of reverse transcription, and positive PCR control reactions. Genes of interest could be classified into the following major categories: transcriptional regulation, osteoblast-related, extracellular matrix markers, cellular adhesion, BMP and SMAD signaling, growth factors, and angiogenic factors. In addition to the above targets, there are other genes included in these arrays which potentially have minor roles in osteogenesis, and hence, do not fit into the above categories.

**Fig. 4:**
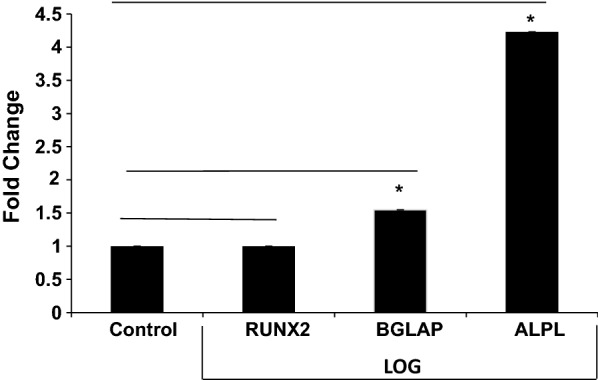
The effects of LOG on RUNX2, BGLAP, and ALPL gene expressions. AD-MSCs were cultured for 21 days in either an osteogenic differentiation media on tissue culture polystyrene (control) or in undifferentiated media on low oxygen graphene (LOG). Data was normalized to 1 by B2M. n = 3; * indicates p < 0.05. Error bars presented as standard deviation

As described earlier, expression profiles of ALPL, BGLAP, and RUNX2 during osteogenesis are commonly used as indicators of cell differentiation and hence, are typically evaluated using single gene PCR reactions following Alizarin red staining [17–19]. Therefore, we examined these gene expressions between human AD-MSCs cultured on LOG in absence of osteogenic differentiation reagents to MSCs cultured on tissue culture substrate in presence of differentiation reagents at day 21. Gene expression on tissue culture substrate was set as the control and that on the LOG surface was set as the test group (Fig. 4). There was a significant (p < 0.05) increase in the expression of ALPL and BGLAP in cells cultured on LOG, confirming osteogenesis under the media conditions described above. RUNX2 was not statistically different on LOG, thereby suggesting the expression levels are similar across these comparisons. To understand and compare the osteogenic signaling mediated by LOG on human AD-MSCs and BM-MSCs, all further comparisons were performed on cells cultured on the LOG surface only.

### Percent of differentially expressed genes suggest early changes in BMMSCs

**Fig. 5:**
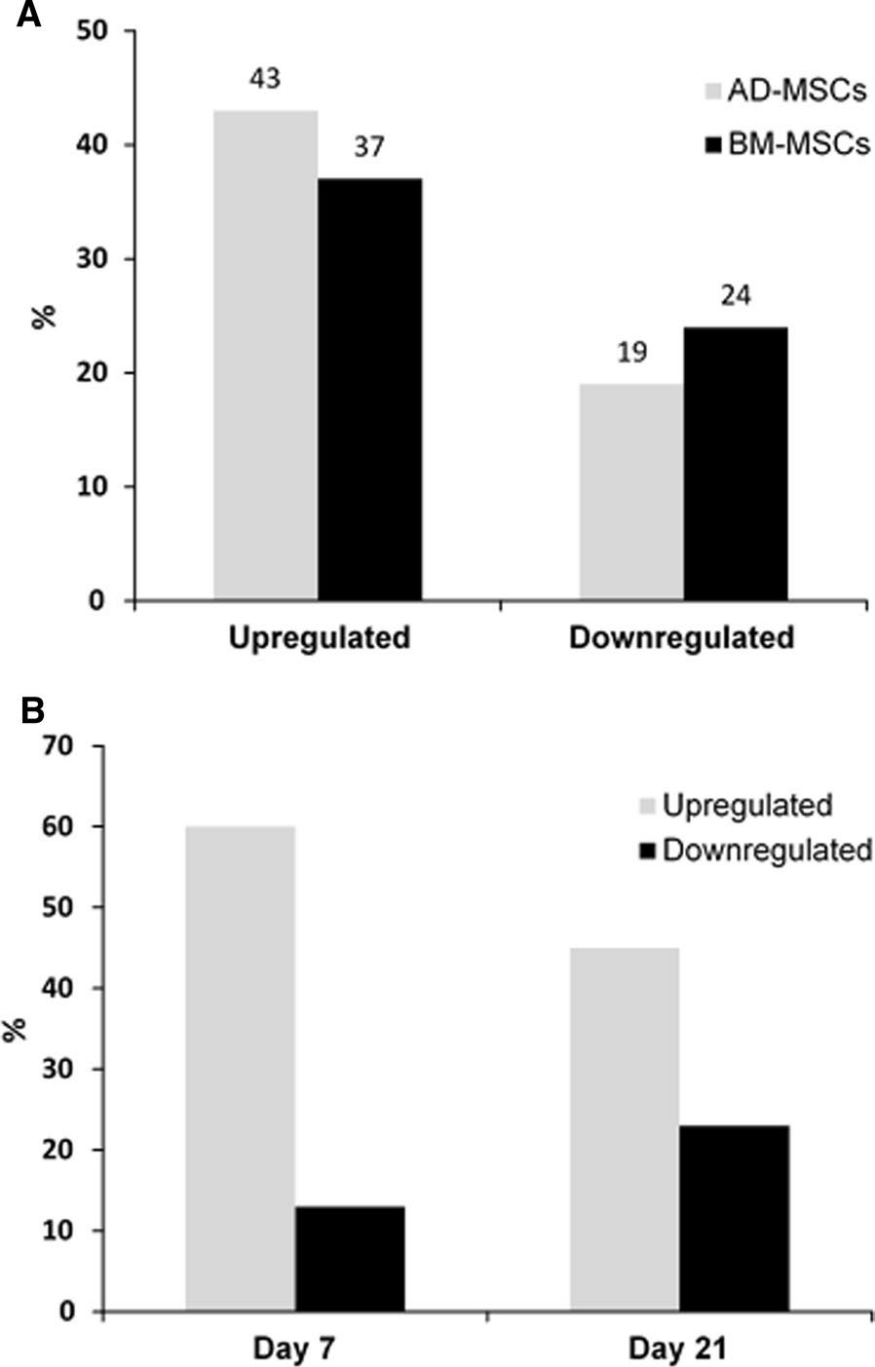
Differentially expressed genes when AD-MSCs and BM-MSCs undergo osteogenesis on LOG. **A** Percentage of significantly changed genes at Day 21 in comparison to its control at Day 7. **B** Percentage of significantly changed genes in BM-MSCs at each time point in comparison to AD-MScs set as the control

Gene expressions patterns of AD-MSCs and BM-MSCs cultured on LOG were over time (day 7 set as control to day 21 set as the test group) within each cell type. Subsequently the patterns were compared between the two cell lines at each time point, thus, resulting in a total of 4 comparisons. Differentially expressed and significantly different genes (p < 0.05) are reported and described in sections below. Over time, the percentage of significantly expressed genes was consistent in both cell types. Comparisons showed that 61–62% of the genes analyzed changed statistically with 37–43% being upregulated while only 19–24% were downregulated (Fig. 5A), complementing the osteogenic response of MSCs on LOG. Interestingly, when day 7 results were compared between the two cell types, 60% of genes were upregulated in BM-MSCs while only 13% were downregulated (Fig. 4B), suggesting upregulation of a higher number of gene targets in BM-MSCs at an earlier time point. Comparatively, at day 21 only 45% of genes were upregulated in BM-MSCs while 23% of genes were downregulated. Across all comparisons, ˂ 10% of genes were unconfirmed, possibly due to low expression or lack of primer annealing, and hence were not detected.

### Distinct transcription factors control osteogenesis of MSCs

We examined the expression of four genes, *DLX5, RUNX2, SOX9*, and *SP7* known to control stem cell fate (Table 1a). As shown, *RUNX2* was significantly upregulated in AD-MSCs, while SOX9 and SP7 were downregulated, suggesting *RUNX2* to be the master regulator in AD-MSCs. Comparatively, in BM-MSCs, all genes were downregulated. Interestingly, when BM-MSCs were compared to AD-MSCs, all transcription factors were upregulated at both time points (Table 2a). The fold changes at day 7 were comparatively more robust to that observed at day 21, suggesting the involvement of all transcription factors triggering osteogenesis at an early time point in BM-MSCs.

**Table 1:** Gene expressions of AD-MSCs and BM-MSCs cultured on LOG between 7 and 21 days

a			
Gene description	Symbol	Fold change (AD-MSCs)	Fold change (BM-MSCs)
**Transcriptional regulation**			
Distal-less homeobox 5	*DLX5*	ND	0.26
Runt-related transcription factor 2	*RUNX2*	1.57	0.78
SRY (sex determining region Y)-box 9	*SOX9*	0.70	0.64
Sp7 transcription factor	*SP7*	0.65	0.21
**Osteoblast-related**			
Alkaline phosphatase, liver/bone/kidney	*ALPL*	2.09	3.43
Bone gamma-carboxyglutamate (gla) protein/osteocalcin	*BGLAP*	1.30	0.50
Phosphate regulating endopeptidase homolog, X-linked	*PHEX*	1.28	1.49
Secreted phosphoprotein 1/osteopontin	*SPP1*	0.30	1.86
**Extracellular matrix markers**			
Biglycan	*BGN*	1.36	1.46
Collagen, type I, alpha 1	*COL1A1*	NC	1.27
Collagen, type I, alpha 2	*COL1A2*	0.81	NC
Collagen, type III, alpha 1	*COL3A1*	1.22	NC
Collagen, type V, alpha 1	*COL5A1*	NC	NC
Collagen, type X, alpha 1	*COL10A1*	NC	2.11
Collagen, type XIV, alpha 1	*COL14A1*	1.31	13.33
Collagen, type XV, alpha 1	*COL15A1*	2.50	11.63
Fibronectin 1	*FN1*	1.49	2.28
**Cellular adhesion**			
Cadherin 11, type 2, OB-cadherin (osteoblast)	*CDH11*	NC	1.62
Intercellular adhesion molecule 1	*ICAM1*	4.69	6.28
Integrin, alpha 1	*ITGA1*	1.62	1.21
Integrin, alpha 2 (CD49B, alpha 2 subunit of VLA-2 receptor)	*ITGA2*	0.49	0.29
Integrin, alpha 3 (antigen CD49C, alpha 3 subunit of VLA-3 receptor)	*ITGA3*	NC	1.66
Integrin, beta 1 (fibronectin receptor, beta polypeptide, antigen CD29 includes MDF2, MSK12)	*ITGB1*	0.66	0.78
Vascular cell adhesion molecule 1	*VCAM1*	2.42	11.13

b			
Gene description	Symbol	Fold change (AD-MSCs)	Fold change (BM-MSCs)
**BMP and SMAD signaling**			
Bone morphogenetic protein 1	*BMP1*	NC	NC
Bone morphogenetic protein 2	*BMP2*	NC	0.25
Bone morphogenetic protein 4	*BMP4*	1.94	2.61
Bone morphogenetic protein 6	*BMP6*	0.15	0.05
Activin A receptor, type I	*ACVR1*	NC	NC
Bone morphogenetic protein receptor, type IA	*BMPR1A*	NC	1.18
Bone morphogenetic protein receptor, type IB	*BMPR1B*	NC	2.04
Bone morphogenetic protein receptor, type II	*BMPR2*	1.19	0.63
SMAD family member 1	*SMAD1*	NC	0.61
SMAD family member 2	*SMAD2*	1.24	NC
SMAD family member 3	*SMAD3*	2.62	2.13
SMAD family member 4	*SMAD4*	NC	0.93
SMAD family member 5	*SMAD5*	1.14	0.88
**Growth factors**			
Epidermal growth factor	*EGF*	0.55	NC
Epidermal growth factor receptor	*EGFR*	1.99	NC
Fibroblast growth factor 1 (acidic)	*FGF1*	0.74	0.69
Fibroblast growth factor 2 (basic)	*FGF2*	0.50	0.78
Fibroblast growth factor receptor 1	*FGFR1*	NC	NC
Fibroblast growth factor receptor 2	*FGFR2*	NC	2.65
Growth differentiation factor 10	*GDF10*	0.18	ND
Insulin-like growth factor 1 (somatomedin C)	*IGF1*	2.53	0.66
Insulin-like growth factor 2 (somatomedin A)	*IGF2*	NC	2.40
Insulin-like growth factor 1 receptor	*IGF1R*	1.44	0.76
Transforming growth factor, beta 1	*TGFB1*	NC	0.76
Transforming growth factor, beta 2	*TGFB2*	1.52	1.53
Transforming growth factor, beta 3	*TGFB3*	2.06	1.80
Transforming growth factor, beta receptor 1	*TGFBR1*	1.32	1.99
Transforming growth factor, beta receptor II (70/80 kDa)	*TGFBR2*	1.57	2.60
Tumor necrosis factor	*TNF*	1.76	ND
**Angiogenic factors**			
Fms-related tyrosine kinase 1 (vascular endothelial growth factor/vascular permeability factor receptor)	*FLT1*	NC	ND
Platelet-derived growth factor alpha polypeptide	*PDGFA*	1.34	NC
Vascular endothelial growth factor A	*VEGFA*	1.40	NC
Vascular endothelial growth factor B	*VEGFB*	1.38	1.40

CT values for each gene were normalized using a housekeeping gene and then the fold changes were calculated by using Day 7 expression as the control and Day 21 expression as the tested group

*NC* No change, *ND* non-detectable

**Table 2:** Gene expressions of BM-MSCs in comparison to AD-MSCs cultured on LOG for either 7 or 21 days

a			
Gene description	Symbol	Day 7	Day 21
		Fold change	Fold change
**Transcriptional regulation**			
Distal-less homeobox 5	*DLX5*	60.69	11.24
Runt-related transcription factor 2	*RUNX2*	7.24	2.24
SRY (sex determining region Y)-box 9	*SOX9*	6.85	3.89
Sp7 transcription factor	*SP7*	9.56	1.87
**Osteoblast-related**			
Alkaline phosphatase, liver/bone/kidney	*ALPL*	0.01	0.01
Bone gamma-carboxyglutamate (gla) protein/osteocalcin	*BGLAP*	3.69	0.88
Phosphate regulating endopeptidase homolog, X-linked	*PHEX*	0.45	0.33
Secreted phosphoprotein 1/osteopontin	*SPP1*	NC	3.06
**Extracellular matrix markers**			
Biglycan	*BGN*	1.93	1.29
Collagen, type I, alpha 1	*COL1A1*	NC	0.59
Collagen, type I, alpha 2	*COL1A2*	NC	NC
Collagen, type III, alpha 1	*COL3A1*	0.45	0.31
Collagen, type V, alpha 1	*COL5A1*	2.08	NC
Collagen, type X, alpha 1	*COL10A1*	13.21	12.50
Collagen, type XIV, alpha 1	*COL14A1*	4.01	25.34
Collagen, type XV, alpha 1	*COL15A1*	0.08	0.22
Fibronectin 1	*FN1*	2.73	2.58
**Cellular adhesion**			
Cadherin 11, type 2, OB-cadherin (osteoblast)	*CDH11*	2.11	2.2
Intercellular adhesion molecule 1	*ICAM1*	0.51	0.43
Integrin, alpha 1	*ITGA1*	2.00	NC
Integrin, alpha 2 (CD49B, alpha 2 subunit of VLA-2 receptor)	*ITGA2*	1.95	0.71
Integrin, alpha 3 (antigen CD49C, alpha 3 subunit of VLA-3 receptor)	*ITGA3*	5.00	4.34
Integrin, beta 1 (fibronectin receptor, beta polypeptide, antigen CD29 includes MDF2, MSK12)	*ITGB1*	1.54	1.14
Vascular cell adhesion molecule 1	*VCAM1*	482.15	1376.38

b			
Gene description	Symbol	Day 7	Day 21
		Fold change	Fold change
**BMP and SMAD signaling**			
Bone morphogenetic protein 1	*BMP1*	1.94	NC
Bone morphogenetic protein 2	*BMP2*	17.92	3.36
Bone morphogenetic protein 4	*BMP4*	0.16	0.13
Bone morphogenetic protein 6	*BMP6*	4.05	NC
Activin A receptor, type I	*ACVR1*	2.26	1.61
Bone morphogenetic protein receptor, type IA	*BMPR1A*	2.23	1.72
Bone morphogenetic protein receptor, type IB	*BMPR1B*	NC	NC
Bone morphogenetic protein receptor, type II	*BMPR2*	4.53	1.50
SMAD family member 1	*SMAD1*	4.14	1.58
SMAD family member 2	*SMAD2*	2.30	1.29
SMAD family member 3	*SMAD3*	NC	NC
SMAD family member 4	*SMAD4*	1.96	1.06
SMAD family member 5	*SMAD5*	3.15	1.50
**Growth factors**			
Epidermal growth factor	*EGF*	4.78	7.73
Epidermal growth factor receptor	*EGFR*	2.38	0.61
Fibroblast growth factor 1 (acidic)	*FGF1*	3.44	1.99
Fibroblast growth factor 2 (basic)	*FGF2*	1.53	1.47
Fibroblast growth factor receptor 1 (acidic)	*FGFR1*	2.37	NC
Fibroblast growth factor receptor 2 (basic)	*FGFR2*	7.57	16.8
Growth differentiation factor 10	*GDF10*	0.02	0.10
Insulin-like growth factor 1 (somatomedin C)	*IGF1*	7.34	1.20
Insulin-like growth factor 2 (somatomedin A)	*IGF2*	34.30	43.71
Insulin-like growth factor 1 receptor	*IGF1R*	5.72	1.89
Transforming growth factor, beta 1	*TGFB1*	2.30	NC
Transforming growth factor, beta 2	*TGFB2*	51.51	32.22
Transforming growth factor, beta 3	*TGFB3*	NC	0.71
Transforming growth factor, beta receptor 1	*TGFBR1*	4.07	3.80
Transforming growth factor, beta receptor II (70/80 kDa)	*TGFBR2*	0.64	0.66
Tumor necrosis factor	*TNF*	ND	NC
**Angiogenic factors**			
Fms-related tyrosine kinase 1 (vascular endothelial growth factor/vascular permeability factor receptor)	*FLT1*	NC	NC
Platelet-derived growth factor alpha polypeptide	*PDGFA*	3.07	1.44
Vascular endothelial growth factor A	*VEGFA*	4.07	1.88
Vascular endothelial growth factor B	*VEGFB*	1.65	NC

CT values for each gene were normalized using a housekeeping gene and then the fold changes were calculated by using AD-MSCs as the control and BM-MSCs as the tested group

*NC* No change, *ND* non-detectable

### Upregulation of osteoblast-related genes confirm osteogenesis in MSCs

Osteoblast differentiation of MSCs is evidenced by the expression of cell-specific markers. We examined the expression of 4 genes, *ALPL*, *BGLAP*, *PHEX*, and *SPP1*, commonly used as markers of osteogenesis (Table 1a). As shown, *ALPL*, *BGLAP* (osteocalcin), and *PHEX* were upregulated in AD-MSCs while *SPP1* was downregulated. Comparatively, in BM-MSCs, in addition to *ALPL* and *PHEX*, *SPP1* was also upregulated, suggesting osteogenesis within 21 days in both cell types. When BM-MSCs were compared to AD-MSCs results were very interesting. Only *BGLAP* was significantly upregulated at day 7, whereas, at day 21 all genes were downregulated with the exception of *SPP1* (Table 2a), suggesting osteogenesis of BM-MSCs at a time point earlier than day 21.

### ECM targets support cell adhesion and differentiation

When cells undergo osteoblast differentiation in both the presence and absence of nanocomposite materials, they express ECM in the form of organic and inorganic molecules. ECM proteins have important roles in cell adhesion and differentiation. ECM proteins trigger signal transduction pathway(s) leading to their differentiation to specific lineages. Once the MSCs are triggered towards differentiation, ECM proteins support the adhesion of the differentiated cells to the substrate, and hence, are required throughout their development. The ECM genes exist as families coding for the various isoforms of the proteins, each form contributing to its function. Here we examined the expression of specific ECM genes including those coding for collagen, fibronectin, and proteoglycan (Tables 1a, 2a). In AD-MSCs, predominantly all genes tested including *BGN*, *COL3A1*, *COL14A1*, *COL15A1* and *FN1* were upregulated while only *COL1A2* was downregulated. Comparatively, in BM-MSCs, all genes were upregulated, with the fold changes much higher than that observed in AD-MSCs. Interestingly when AD-MSCs were compared to BM-MSCs at day 7, all genes except *COL3A1* and *COL15A1* were upregulated (Table 2a). Taken together, these data suggest that the specific genes encoding ECM proteins support the adherence and osteogenesis of MSCs from both the sources.

### Relatively robust upregulation of genes encoding for cell adhesion proteins

MSCs adhere to a given surface and express ECM, and subsequently relay extracellular signals to the nucleus for osteogenic differentiation and communication. MSCs are adherent cells and hence, cell adhesion proteins are required for the attachment and cell development. In this study, 7 cellular adhesion genes were examined (Table 1a). In AD-MSCs, *ICAM1*, *ITGA1*, and *VCAM1* were all upregulated while only *ITGA2* and *ITGB1* were downregulated. Similar patterns of expression were observed in BM-MSCs with upregulation of *CDH11*, *ICAM1*, *ITGA1*, *ITGA3*, and *VCAM1*, and downregulation of only *ITGA2* and *ITGB1*. Comparatively, when AD-MSCs were compared to BM-MSCs, *CDH11*, *ITGA1*, *ITGA2*, *ITGA3*, *ITGB1* were all upregulated at day 7. Similar expression patterns were observed at day 21, with the exception of *ITGA2*. Noteworthy is the 482.15- and 1376.38-fold upregulation of *VCAM1* at days 7 and 21, respectively (Table 2a). Results confirm that LOG surface provides an optimal substrate for cells to adhere, communicate, differentiate, and maintain their function.

### BMP/SMAD-mediated osteogenesis in MSCs

BMP-SMAD signaling is one of the major pathways that the cells use when they undergo osteoblast differentiation. BMP and the SMAD families of genes consist of multiple isoforms, majority of which were represented in these arrays. We examined 4 BMPs, 4 BMP receptors (BMPRs) and 5 SMAD isoforms (Table 1b). In AD-MSCs, only *BMP4* and *BMPR2* were upregulated while *BMP6* was downregulated. Similarly, all SMAD isoforms including, *SMAD2*, *SMAD3*, and *SMAD5* were upregulated suggesting that osteogenesis is potentially mediated by BMP/SMAD signaling. Comparatively, in BM-MSCs, only *BMP4*, *BMPR1A* and *BMPR1B* were upregulated, while *BMP2*, *BMP6* and *BMPR2* were downregulated. Of the SMAD genes tested in BM-MSCs, only *SMAD3* was upregulated while *SMAD1*, *SMAD4*, and *SMAD5* were downregulated. Interestingly when AD-MSCs were compared with BM-MSCs, patterns of expression suggestive of BMP/SMAD signaling in BM-MSCs were observed (Table 2b). At Day 7, *BMP1*, *BMP2*, and *BMP6* were upregulated while only *BMP4* was downregulated. At Day 21, however, only *BMP2* was upregulated and *BMP4* was downregulated. Additionally, all BMPRs and SMAD genes demonstrated robust upregulation at both time points. Results further support that similar to AD-MSCs, osteogenesis of BM-MSCs is also mediated by BMP/SMAD signaling, and potentially occurs at a time point earlier than day 21.

### TGFβ family members are involved in osteogenesis

In addition to the BMPs, growth factors including EGF, FGF, IGF and their corresponding receptors are also involved in bone tissue healing, regeneration, and cell differentiation. In this study we examined 10 growth factors and 6 growth factor receptors (Table 1b). In AD-MSCs, *IGF1*, *TGFB2*, *TGFB3*, and *TNF* were upregulated while *EGF*, *FGF1*, *FGF2*, and *GDF10* were downregulated. All growth factor receptor genes, including *EGFR*, *IGF1R*, *TGFBR1*, and *TGFBR2* were upregulated. Comparatively, in BM-MSCs, *IGF2*, *TGFB2*, and *TGFB3* were upregulated while *FGF1*, *FGF2*, *IGF1*, *TGFB1* were downregulated. Correspondingly, the growth factor receptors including, *FGFR2*, *TGFBR1*, and *TGFBR2* were upregulated, while only *IGF1R* was downregulated. Fold expression changes between the two cell types were very striking. At day 7, only *GDF10* was downregulated, while at Day 21 both *GDF10* and *TGFB3* were downregulated (Table 2b). All other growth factors including *EGF*, *FGF1*, *FGF2*, *IGF1*, *IGF2*, *TGFB1*, and *TGFB2* were upregulated at both time points with significantly higher changes in *TGFB2*. Similarly, all growth factor receptors were upregulated at both time points with the exception of *TGFBR2* and *EGFR* which was downregulated at day 21 only, suggesting the potential involvement of the TGF beta family of genes and their corresponding receptors, mediated osteogenesis of MSCs on LOG.

### Significant upregulation of angiogenic factors at all-time points

Angiogenesis is closely entwined with osteoblast differentiation. Formation of new blood vessels along with maintenance of new and old blood vessels are both coupled with osteogenesis [28]. In AD-MSCs, *PDGFA*, *VEGFA*, and *VEGFB* were all upregulated (Table 1b). Comparatively, in BM-MSCs, only *VEGFB* was upregulated. There was no downregulation of any angiogenic markers at any time point (Table 2b). Interestingly, when AD-MSCs were compared to BM-MSCs, *PDGFA*, *VEGFA* and *VEGFB*, all displayed significant upregulation at both time points, suggesting the angiogenic potential of MSCs in presence of LOG.

### Cytoscape analysis demonstrates potential gene interactions at the protein level

**Fig. 6:**
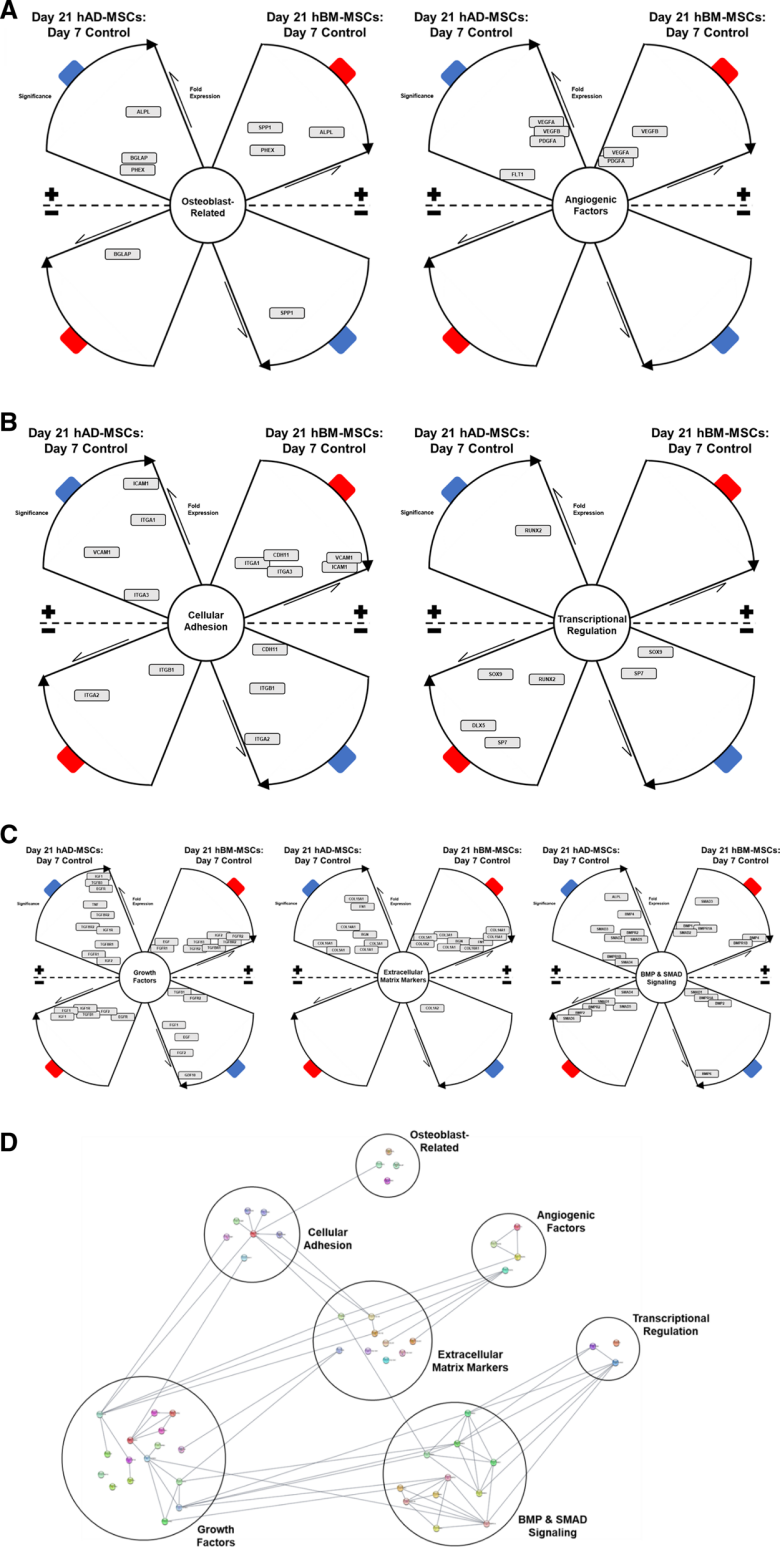
Propellor plots depicting potential gene targets and corresponding protein interactions. Cytoscape analyses illustrating differentially expressed genes related to Osteoblast and Angiogenic factors (**A**), Cellular adhesion and transcriptional regulation (**B**), and Growth factors, ECM markers and members of BMP/SMAD signaling (**C**) to be the key targets involved in osteogenic differentiation of human AD and BM-derived MSCs. In all analyses, day 7 expression was set as control and day 21 was the treated group. The cell types are color coded, with AD-MSCs (Blue) and BM-MSCs (Red), and the plot arcs with increasing significance moving clockwise and a decrease is represented as anticlockwise. **D** Connectively plot for target genes sorted based on established functional groups. Lines linking nodes indicate relationships between associated proteins as annotated by STRING application software within the Cytoscape platform. Interconnective links within and between functional groupings can be observed

The significant changes described above were translated to potential signaling pathways that the MSCs undergo during osteogenesis on LOG. Using Cytoscape, an open source software platform, we visualized the molecular interaction between the gene targets and their potential proteins (Fig. 6). The propellor plots shown in this figure demonstrate a significantly high number of gene/protein targets from the ECM markers and the TGFβ/BMP/SMAD pathways to have a role in osteogenic differentiation of MSCs on LOG. The plots confirm our earlier report that ECM proteins play an important role in the adhesion and subsequent differentiation of MSCs on LOG [11]. Data presented here demonstrates that osteogenesis of both the AD and BM MSCs is potentially mediated by the TGFβ/BMP/SMAD signaling.

### Immunofluorescence confirms cytoskeletal organization and distinct ECM protein expression of BM-MSCs

**Fig. 7:**
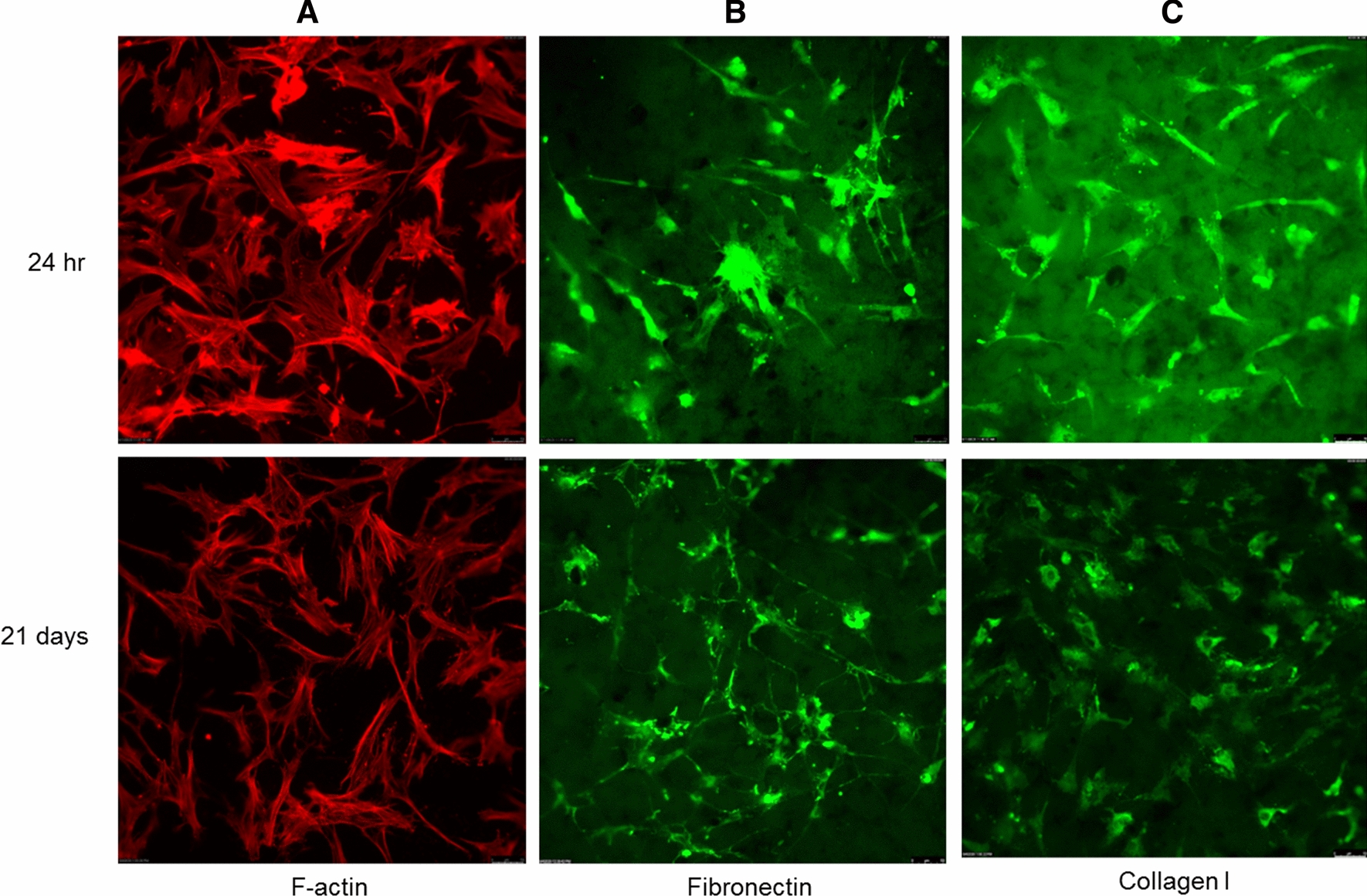
Immunofluorescence assays. Assays were performed to assess cytoskeletal organization of BMMSCs using F-actin (**A**) and expression of specific ECM proteins (**B**, **C**) during cell adhesion at 24 h and differentiation at day 21

Phalloidin F-actin staining illustrates cytoskeletal organization and cell adhesion and hence, is a powerful tool to show cell attachment onto biomaterials. We have previously demonstrated cytoskeletal health and integrity of AD-MSCs on LOG [11]. In this study, we confirmed the cytoskeletal organization of BM-MSCs on LOG (Fig. 7A) at 24 h and 21 days i.e. at the adhesion and differentiation time points. Even though the cytoskeletal integrity appears to be maintained at both time points, subjectively, cells appear discretely localized and clustered at the 24 h time point, supporting our earlier data that cell attachment to LOG surface is not random. Subsequently, the expression and localization of fibronectin and collagen 1 evaluated at the same time points confirm that the two ECM proteins have roles in cell adhesion and differentiation on LOG surface (Fig. 7B, C). These data complement our report on the behavior of AD-MSCs [11]. Subjective evaluation of fibronectin and collagen at 24 h and at day 21 suggests higher expression and a relatively more discrete pattern of expression at 24 h. Even though BM-MSCs express these ECM proteins at day 21, cells are sparse and the expression is weak, suggesting that cells undergo osteogenesis presumably at a time point earlier than day 21. The significant down regulation of genes at day 21 as described in the above sections complements this observation. A future study that investigates an osteogenic response and the expression of ECM proteins in BM-MSCs, between 24 h and day 21 is needed to confirm this data.

## Discussion

In this study, we present data comparing the osteogenic behavior of human adipose- and bone marrow-derived MSCs in presence of LOG using in vitro osteogenesis assays and genetic profiling. To our knowledge, this is the first study to compare changes in gene expressions when MSCs from two distinct tissue sources undergo spontaneous (without any chemical induction) osteogenic differentiation in presence of graphene. Additionally, this is the first study reporting simultaneous genetic profiling of a panel of genes involved in cell adhesion, production of ECM, osteoblast differentiation, and ossification. Changes in gene expression provides a mechanistic overview of the key targets that are potentially involved in graphene—mediated osteogenic signaling of MSCs. This data strengthens the use of MSCs + graphene surfaces as scaffold components for bone tissue engineering.

When placed in an osteogenic environment, MSCs have the potential to differentiate into osteoblasts (bone cells). This commitment is regulated by osteoblast-associated transcription factors (DLX5, RUNX2, SP7, SOX9), adhesion molecules (integrins β1/ITGB1), and extracellular matrix proteins (ECM) (fibronectin, collagen I) [29]. During differentiation, cells generate tissue-specific ECM, express ALPL, BGLAP, and SPP1, and undergo bone cell development. Renowned osteogenic pathways include the WNT/β-catenin and bone morphogenetic protein (BMP)/transforming growth factor beta (TGFβ) pathways. The WNT proteins activate at least three distinct intracellular signaling cascades important for osteogenic differentiation [30]. Some studies suggest cross talk between WNT, MAPK, and TGFβ signaling when MSCs undergo osteogenesis [31]. On the other hand, BMP/TGFβ signaling has been thoroughly reviewed in bone development which functions through both canonical (SMAD dependent) and non-canonical (SMAD independent) pathways, thereby mediating transcription [32, 33]. Additionally, the Hedgehog (Hh) and Notch pathways are suggested to affect cell osteogenesis, but their exact role is unknown [34–38]. It is thus evident that the osteogenic signaling pathways are complex and involve a coordinated action of multiple genes and their corresponding protein factors.

Bone marrow and adipose tissue are the most common MSC resources [39–44]. Although bone marrow is considered the richest source of MSCs in humans and animals, fat-derived MSCs are preferred in many clinics or in basic research projects, because the tissue harvest is relatively easy, less invasive, and not associated with patient morbidity [45–48]. Although MSCs isolated from the two tissue sources adhere to tissue culture polystyrene surface, show similar expression patterns of cluster-of-differentiation markers, morphology, and trilineage differentiation potential in vitro [43], they might exhibit differences in their lineage-specific features and overall functionality. These variations have been reported in presence and absence of biomaterials [49–51]. For instance, we expect the BM-MSCs to exhibit an increased potential towards osteogenesis as studies show that in the absence of any biomaterial, AD and BM-MSCs undergo osteogenesis by different signaling pathways [52]. Therefore, it is possible that their interaction with materials might affect this process, and BM-MSCs may not be the optimal cell type to use. For example, it was found that osteogenic induction of BM-MSCs was mediated by the p38 MAPK pathway, while AD-MSCS involved the p44/42 MAPK pathway [25]. Contrary to this report, other studies have found that PDGF enhances osteogenesis of AD-MSCs, but not BM-MSCs [53]. Hence, for an efficacious stem cell therapy, it is important to study a specific cell type in context of a given biomaterial in vitro and in vivo.

The AD and BM-MSCs undergo osteogenesis on tissue culture polystyrene surface within 21–28 days in conditions of chemical induction. Osteogenesis is a programmed process which is accompanied by dynamic changes in the expression profiles of osteoblast-related genes. Early markers of osteogenesis are expressed as early as 7–10 days post induction [43, 54, 55], and completed within 21–28 days concomitant with the expression of late osteogenic markers. As a result, we examined differences in gene expressions at days 7 and 21 when AD and BM-MSCs are cultured on graphene. Since osteoblast development from MSCs is recognized by mineralization, which can be visualized by alizarin red staining and quantitated by elution of the red color, we found that both AD and BM-MSCs were differentiated by 21 days when chemically induced on tissue culture polystyrene and when non-chemically induced on graphene (Fig. 2). Osteogenic differentiation under these media conditions was confirmed by the increase in ALPL and BGLAP expression in AD-MSCs cultured on graphene relative to the tissue culture polystyrene surface (Fig. 4). Lower mineralization was observed when AD and BM-MSCs were simultaneously exposed to osteo-chemical inducers and graphene. Although we confirmed the cells were viable (data not shown), it is possible that the combination triggers distinct signal transduction pathway(s) [26], which is beyond the scope of this study.

Graphene-based nanomaterials have been recognized as successful components of bone tissue engineering scaffolds [4, 56–60]. Studies recognize graphene as a delivery vehicle to control the release and dosing of potent BMP2 treatments for endogenous stem cell activation [61–64], or as a nanomaterial which by virtue of its physicochemical properties creates an osteogenic environment triggering both the endogenous and exogenous stem cells to undergo osteogenesis. Graphene studies consistently show osteogenesis of various MSCs sources [17–19, 65–78], which is generally supported by mineralization stains and upregulation of bone-specific markers i.e. RUNX2, ALPL, BMP2, BGLAP, SPP1, and COL I. These studies demonstrate the end result of osteogenic differentiation but lack understanding of how the signaling process occurs. Most recently, it was shown that human dental pulp MSCs in the presence of graphene achieved osteogenesis via the integrin/focal adhesion kinase axis, thereby signaling SMAD phosphorylation, RUNX2 transcription, and production of BGLAP and SPP1 proteins [76]. It is suggested that the carbon arrangement of graphene and its derivatives mimics an organic bone ECM microenvironment, whereby stem cells can attach, proliferate and ultimately differentiate under the appropriate cues [11]. As a result, cell adhesion is the initiating event, since without this attachment, cells have a limited opportunity to produce their ECM and be signaled into differentiation. Subsequently, extracellular ligands can bind to cell surface receptors and relay signals into the nucleus for transcriptional activation that commits osteogenic differentiation. It is possible that by virtue of its planar structure, the low oxygen content form of graphene makes cells accessible to the graphene surface, potentially providing guidance and an appropriate topography to attach, cluster, and thereby differentiate into osteogenic lineage. Our data supports this theory as both MSC sources on the low oxygen graphene either maintained or positively expressed several adhesion (i.e. CDH11, ICAM1, ITGA1, ITGA3, VCAM1) and ECM (i.e. BGN, COL1A1, COL3A1, COL10A1, COL14A1, COL15A1, FN1) genes over time.

In addition to osteogenesis, there are some reports demonstrating the angiogenic effect of graphene nanomaterials. Park et al. showed that rGO flakes incorporated with MSC spheroids stimulated the expression of angiogenic growth factors, including VEGF, HGF, and FGF2 [79]. Similarly, it was found that graphene-based biomaterials not only differentiated cells into osteoblasts, but simultaneously increased other angiogenic markers, namely von Willibrand factor (vWF) and angiopoietin-1 (ang-1) [74]. Other studies have found that low concentrations of graphene derivatives (up to 100 ng/mL^−1^) triggers a pro-angiogenic environment via Akt and nitric oxide signaling of endothelial cells [80].

Our data supports a recent study that demonstrated graphene-mediated osteogenesis via BMP/SMAD pathways [81]. However, for the first time, we provide information on the various protein isoforms belonging to these families. We not only show expressions of osteoblast-related genes, but also the coordinated involvement of cellular adhesion molecules, ECM, growth factors, and angiogenic factors which are all necessary for osteogenic signaling, maintenance, and survival. Additionally, no study has completed a head-to-head comparison of different MSC sources on graphene. The current literature primarily studies MSCs associated with mineralized tissues (i.e., bone marrow, dental pulp, and periodontal ligament) [18, 19, 70, 71, 73, 74, 76–78], with only one other research group identifying osteogenesis of AD-MSCs on graphene [69]. Our study recognizes that AD-MSCs may achieve osteogenesis slower than BM-MSCs but are still a valid and feasible resource for bone healing and repair.

In this study, we demonstrate that both AD and BM-MSCs undergo osteogenesis on graphene surfaces which is mediated by multiple transcription factors in addition to RUNX2, the most commonly reported in all the studies described above. The transcriptional regulation appears to be controlled by RUNX2 and DLX5. This is a novel finding and suggests that the action of RUNX2 and DLX5 may be synergistic in the osteogenic behavior of BM-MSCs in presence of graphene. DLX5 and RUNX2 has been reported to have a significant role in early bone development, by mediating intramembranous and endochondral ossification, respectively [82–84]. Additionally, comparative assessment indicates that the osteogenic commitment of BM-MSCs occurs at a time point earlier than day 21. This is significant and can greatly affect in vitro and in vivo studies using graphene nanomaterials. Other novel targets identified in this study include *PHEX* (phosphate‐regulating gene with homologies to endopeptidase on the X chromosome), an osteoblast-related gene that when inactive, leads to excessive phosphate wasting and consequently causes rickets [85]. In contrast to bone, we also examined chondrocyte-related genes, namely SOX9 and COMP (cartilage oligomeric matrix protein, data not shown) which were downregulated in both cell types over time. Finally, our data supports that culturing MSCs on graphene upregulates angiogenic markers, *VEGF* and *PDGF*. Interestingly, *PDGF* bridges the osteogenic and angiogenic pathways by freeing MSCs from blood vessels and positively regulating *VEGF* signaling [86]. This data suggests that graphene surfaces could simultaneously attract blood vessel ingrowth when implanted in vivo.

## Conclusion

This study investigates the genetic responses when MSCs undergo osteogenesis on graphene. Graphene genetically supports osteogenesis of MSCs by multiple transcription factors, extracellular matrix production, adhesion molecules, growth factor signaling, and angiogenic markers. Additionally, we provide this information from various MSCs sources, which have similar outcomes on graphene, but different mechanisms to osteoblast-development. These results provide optimism that exogenous MSCs implanted with graphene materials could support new bone development in future animal models and human clinical trials.

## Data Availability

All data generated or analyzed during this study are included in the article.

## References

[1] Atala A (2004). Tissue engineering and regenerative medicine: concepts for clinical application. Rejuvenation Res.

[2] Hong G (2015). Carbon Nanomaterials for biological imaging and nanomedicinal therapy. Chem Rev.

[3] Georgakilas V (2012). Functionalization of graphene: covalent and non-covalent approaches, derivatives and applications. Chem Rev.

[4] Mao HY (2013). Graphene: promises, facts, opportunities, and challenges in nanomedicine. Chem Rev.

[5] Yang K (2013). Preparation and functionalization of graphene nanocomposites for biomedical applications. Nat Protoc.

[6] Majeed W (2017). The role of surface chemistry in the cytotoxicity profile of graphene. J Appl Toxicol.

[7] Bourdo SE (2017). Physicochemical characteristics of pristine and functionalized graphene. J Appl Toxicol.

[8] Kalbacova M (2010). Graphene substrates promote adherence of human osteoblasts and mesenchymal stromal cells. Carbon.

[9] Elkhenany H (2015). Graphene supports in vitro proliferation and osteogenic differentiation of goat adult mesenchymal stem cells: potential for bone tissue engineering. J Appl Toxicol.

[10] Elkhenany H (2017). Graphene nanoparticles as osteoinductive and osteoconductive platform for stem cell and bone regeneration. Nanomedicine.

[11] Newby SD (2020). Functionalized graphene nanoparticles induce human mesenchymal stem cells to express distinct extracellular matrix proteins mediating osteogenesis. Int J Nanomedicine.

[12] Perez RA (2015). Therapeutically relevant aspects in bone repair and regeneration. Mater Today.

[13] Prasadh S, Suresh S, Wong R (2018). Osteogenic potential of graphene in bone tissue engineering scaffolds. Materials (Basel).

[14] Phinney DG (1999). Donor variation in the growth properties and osteogenic potential of human marrow stromal cells. J Cell Biochem.

[15] Katsara O (2011). Effects of donor age, gender, and in vitro cellular aging on the phenotypic, functional, and molecular characteristics of mouse bone marrow-derived mesenchymal stem cells. Stem Cells Dev.

[16] Zaim M (2012). Donor age and long-term culture affect differentiation and proliferation of human bone marrow mesenchymal stem cells. Ann Hematol.

[17] Lee JH (2015). Reduced graphene oxide-coated hydroxyapatite composites stimulate spontaneous osteogenic differentiation of human mesenchymal stem cells. Nanoscale.

[18] Xie H (2015). Two and three-dimensional graphene substrates to magnify osteogenic differentiation of periodontal ligament stem cells. Carbon.

[19] Yang X (2019). Effects of graphene oxide and graphene oxide quantum dots on the osteogenic differentiation of stem cells from human exfoliated deciduous teeth. Artif Cells Nanomed Biotechnol.

[20] Wofford A (2020). Human fat-derived mesenchymal stem cells xenogenically implanted in a rat model show enhanced new bone formation in maxillary alveolar tooth defects. Stem Cells Int.

[21] Mueller O, Lightfoot S, Schroeder A (2004). RNA integrity number (RIN)-standardization of RNA quality control. Agilent Appl Note.

[22] Osteogenesis H. Profiler ™ PCR array; 2021.

[23] Gentile F (2010). Cells preferentially grow on rough substrates. Biomaterials.

[24] Tang LA (2012). Highly wrinkled cross-linked graphene oxide membranes for biological and charge-storage applications. Small.

[25] Elkhenany H (2016). Impact of the source and serial passaging of goat mesenchymal stem cells on osteogenic differentiation potential: implications for bone tissue engineering. Journal of animal science and biotechnology.

[26] Langenbach F, Handschel J (2013). Effects of dexamethasone, ascorbic acid and β-glycerophosphate on the osteogenic differentiation of stem cells in vitro. Stem Cell Res Ther.

[27] Kukurba KR, Montgomery SB (2015). RNA sequencing and analysis. Cold Spring Harb Protoc.

[28] Grosso A (2017). It takes two to tango: coupling of angiogenesis and osteogenesis for bone regeneration. Front Bioeng Biotechnol.

[29] Huang W (2007). Signaling and transcriptional regulation in osteoblast commitment and differentiation. Front Biosci.

[30] Houschyar KS (2018). Wnt pathway in bone repair and regeneration—what do we know so far. Front Cell Dev Biol.

[31] Gregory CA (2005). How Wnt signaling affects bone repair by mesenchymal stem cells from the bone marrow. Ann N Y Acad Sci.

[32] Chen G, Deng C, Li Y-P (2012). TGF-β and BMP signaling in osteoblast differentiation and bone formation. Int J Biol Sci.

[33] Guo X, Wang X-F (2009). Signaling cross-talk between TGF-beta/BMP and other pathways. Cell Res.

[34] Long F (2004). Ihh signaling is directly required for the osteoblast lineage in the endochondral skeleton. Development.

[35] Huelsken J, Birchmeier W (2001). New aspects of Wnt signaling pathways in higher vertebrates. Curr Opin Genet Dev.

[36] Engin F (2008). Dimorphic effects of Notch signaling in bone homeostasis. Nat Med.

[37] Wozney JM (1988). Novel regulators of bone formation: molecular clones and activities. Science.

[38] Gazzerro E, Canalis E (2006). Bone morphogenetic proteins and their antagonists. Rev Endocr Metab Disord.

[39] Caplan AI (2005). Review: mesenchymal stem cells: cell-based reconstructive therapy in orthopedics. Tissue Eng.

[40] Caplan AI (2007). Adult mesenchymal stem cells for tissue engineering versus regenerative medicine. J Cell Physiol.

[41] Caplan AI, Dennis JE (2006). Mesenchymal stem cells as trophic mediators. J Cell Biochem.

[42] De Miguel MP (2012). Immunosuppressive properties of mesenchymal stem cells: advances and applications. Curr Mol Med.

[43] Dominici M (2006). Minimal criteria for defining multipotent mesenchymal stromal cells. The International Society for Cellular Therapy position statement. Cytotherapy.

[44] Pittenger MF (2008). Mesenchymal stem cells from adult bone marrow. Methods Mol Biol.

[45] Al-Nbaheen M (2013). Human stromal (mesenchymal) stem cells from bone marrow, adipose tissue and skin exhibit differences in molecular phenotype and differentiation potential. Stem Cell Rev.

[46] Barry FP, Murphy JM (2004). Mesenchymal stem cells: clinical applications and biological characterization. Int J Biochem Cell Biol.

[47] Bieback K (2008). Comparing mesenchymal stromal cells from different human tissues: bone marrow, adipose tissue and umbilical cord blood. Biomed Mater Eng.

[48] Yoshimura H (2007). Comparison of rat mesenchymal stem cells derived from bone marrow, synovium, periosteum, adipose tissue, and muscle. Cell Tissue Res.

[49] Im GI, Shin YW, Lee KB (2005). Do adipose tissue-derived mesenchymal stem cells have the same osteogenic and chondrogenic potential as bone marrow-derived cells?. Osteoarthritis Cartilage.

[50] Kaivosoja E (2012). Chemical and physical properties of regenerative medicine materials controlling stem cell fate. Ann Med.

[51] Rath SN (2016). Adipose- and bone marrow-derived mesenchymal stem cells display different osteogenic differentiation patterns in 3D bioactive glass-based scaffolds. J Tissue Eng Regen Med.

[52] Alajez N (2018). Which stem cells to choose for regenerative medicine application: Bone marrow and adipose tissue stromal stem cells & #8211; similarities and differences. J Nat Sci Med.

[53] Hung BP (2015). Platelet-derived growth factor BB enhances osteogenesis of adipose-derived but not bone marrow-derived mesenchymal stromal/stem cells. Stem Cells.

[54] Shui C (2003). Changes in Runx2/Cbfa1 expression and activity during osteoblastic differentiation of human bone marrow stromal cells. J Bone Miner Res.

[55] Peng L (2008). Comparative analysis of mesenchymal stem cells from bone marrow, cartilage, and adipose tissue. Stem Cells Dev.

[56] Bressan E (2014). Graphene based scaffolds effects on stem cells commitment. J Transl Med.

[57] Byun J (2015). Emerging frontiers of graphene in biomedicine. J Microbiol Biotechnol.

[58] Ding X, Liu H, Fan Y (2015). Graphene - based materials in regenerative medicine. Adv Healthcare Mater.

[59] Goenka S, Sant V, Sant S (2014). Graphene-based nanomaterials for drug delivery and tissue engineering. J Control Release.

[60] Wick P, Louw-Gaume AE, Kucki M (2014). Classification framework for graphene-based materials. Angew Chem Int Ed.

[61] La W-G (2014). Bone morphogenetic protein-2 for bone regeneration – Dose reduction through graphene oxide-based delivery. Carbon.

[62] Zhong C (2017). Continuous release of bone morphogenetic protein-2 through nano-graphene oxide-based delivery influences the activation of the NF-κB signal transduction pathway. Int J Nanomed.

[63] Fu C (2017). Enhancing cell proliferation and osteogenic differentiation of MC3T3-E1 pre-osteoblasts by BMP-2 delivery in graphene oxide-incorporated PLGA/HA biodegradable microcarriers. Sci Rep.

[64] Wang Q (2020). Molecular mechanisms of interactions between BMP-2 and graphene: Effects of functional groups and microscopic morphology. Appl Surf Sci.

[65] Nayak TR (2011). Graphene for controlled and accelerated osteogenic differentiation of human mesenchymal stem cells. ACS Nano.

[66] Crowder SW (2013). Three-dimensional graphene foams promote osteogenic differentiation of human mesenchymal stem cells. Nanoscale.

[67] Kim J (2013). Graphene-incorporated chitosan substrata for adhesion and differentiation of human mesenchymal stem cells. J Mater Chem B.

[68] Lee JH (2015). Enhanced osteogenesis by reduced graphene oxide/hydroxyapatite nanocomposites. Sci Rep.

[69] Lyu CQ (2015). Induction of osteogenic differentiation of human adipose-derived stem cells by a novel self-supporting graphene hydrogel film and the possible underlying mechanism. ACS Appl Mater Interfaces.

[70] Mo X (2016). Enhanced stem cell osteogenic differentiation by bioactive glass functionalized graphene oxide substrates. J Nanomater.

[71] Zhou Q (2016). Bioactivity of periodontal ligament stem cells on sodium titanate coated with graphene oxide. Sci Rep.

[72] Hermenean A (2017). Chitosan-graphene oxide 3D scaffolds as promising tools for bone regeneration in critical-size mouse calvarial defects. Sci Rep.

[73] Radunovic M (2017). Graphene oxide enrichment of collagen membranes improves DPSCs differentiation and controls inflammation occurrence. J Biomed Mater Res A.

[74] Shie MY (2017). Synergistic acceleration in the osteogenic and angiogenic differentiation of human mesenchymal stem cells by calcium silicate-graphene composites. Mater Sci Eng C Mater Biol Appl.

[75] Kim J (2018). Enhanced osteogenic commitment of murine mesenchymal stem cells on graphene oxide substrate. Biomater Res.

[76] Xie H (2019). Graphene-induced osteogenic differentiation is mediated by the integrin/FAK axis. Int J Mol Sci.

[77] Di Carlo R (2020). Osteoblastic differentiation on graphene oxide-functionalized titanium surfaces: an in vitro study. Nanomaterials (Basel).

[78] Zhang J (2020). 3D bioprinting of graphene oxide-incorporated cell-laden bone mimicking scaffolds for promoting scaffold fidelity, osteogenic differentiation and mineralization. Acta Biomater.

[79] Park J (2015). Graphene potentiates the myocardial repair efficacy of mesenchymal stem cells by stimulating the expression of angiogenic growth factors and gap junction protein. Adv Funct Mater.

[80] Mukherjee S (2015). Graphene oxides show angiogenic properties. Adv Healthcare Mater.

[81] Mahmood M (2013). Role of carbonaceous nanomaterials in stimulating osteogenesis in mammalian bone cells. J Mater Chem B.

[82] Tadic T (2002). Overexpression of Dlx5 in chicken calvarial cells accelerates osteoblastic differentiation. J Bone Miner Res.

[83] Holleville N (2003). BMP signals regulate Dlx5 during early avian skull development. Dev Biol.

[84] McGee-Lawrence ME (2014). Runx2 is required for early stages of endochondral bone formation but delays final stages of bone repair in Axin2-deficient mice. Bone.

[85] Francis F (1995). A gene (PEX) with homologies to endopeptidases is mutated in patients with X-linked hypophosphatemic rickets. Nat Genet.

[86] Caplan A, Correa D (2011). PDGF in bone formation and regeneration: new insights into a novel mechanism involving MSCs. J Orthopaed Res.

